# Views on volunteering in mental health: a focus group study with mental health professionals and volunteers in Portugal

**DOI:** 10.1007/s44192-023-00038-1

**Published:** 2023-06-22

**Authors:** Mariana Pinto da Costa, Jaime Oliveira

**Affiliations:** 1 Institute of Psychiatry, Psychology and Neuroscience, King’s College London, London, UK; 2 Institute of Biomedical Sciences Abel Salazar, University of Porto, Porto, Portugal; 3 Institute of Public Health of the University of Porto, Porto, Portugal; 4 Center for Health Technology and Services Research (CINTESIS@RISE), Aveiro, Portugal; 5 Department of Education and Psychology, University of Aveiro, Campus Universitário de Santiago, 3810-193 Aveiro, Portugal

**Keywords:** Volunteering, Volunteers, Mental health professionals, Mental health, Qualitative secondary analysis, Thematic analysis

## Abstract

**Introduction:**

Volunteering has reported health benefits. However, little is known in Portugal about the views of mental health professionals and volunteers on volunteering in mental health care.

**Methods:**

A qualitative secondary analysis of data from six focus groups with 28 participants was conducted in order to explore and compare the perspectives on volunteering in mental health of two stakeholders: mental health professionals and volunteers in Portugal.

**Results:**

Four main themes arose: the nature of the volunteering relationship; volunteering has multiple aims; technology has potential for volunteering; and volunteering has its challenges. Although there were mostly commonalities between their views, some variability suggested that different stakeholders may consider different aspects of volunteering differently. Overall, stakeholders called for structured recruitment and support, training, defining boundaries and fighting the stigma of mental illness.

**Conclusion:**

Despite the lack of volunteering tradition in mental health care in Portugal, volunteering programmes were perceived as an important resource for patients with mental illness.

**Supplementary Information:**

The online version contains supplementary material available at 10.1007/s44192-023-00038-1.

## Introduction

Volunteering has been defined as a ‘non-paid, voluntary activity that benefits others’ [1, 2]. Whilst the focus is on the recipient, benefits on volunteer’s health outcomes such as self-esteem [3], happiness [4], mortality [5] and overall better mental and physical health, have been reported [6, 7].

Volunteering programmes in mental health care usually range from befriending (i.e., a supportive social relationship between the volunteer and the patient) to peer support (i.e., where a volunteer with personal experience of mental illness supports another person with mental illness) [8]. These aim to help patients to engage with their communities [9] by offering a spectrum of volunteering relationship practices, where at one end is a more structured intervention and at the other end, there is a closer resemblance to a naturally occurring friendship [10]. In the centre of this spectrum [10], combining elements of both personal and professional relationships, a model has been proposed [11], which acknowledges reciprocal disclosure and joint activities, but retains a power imbalance with the befriender taking a prescriptive role in steering conversations.

Portugal in 2013 was in third to last place for the percentage of Europeans that perform regular and occasional volunteering work [12]. Portugal witnessed its population participation in volunteering diminish from 19% in 1990 to 12% in 2011, which is half of the European Union’s (EU) average percentage. In fact, with respect to the population that volunteers regularly, Portugal stands at 3%, while the EU’s percentage stands at 11% [12]. Furthermore, while in some European countries volunteering has been having a visible economic value (e.g. 4.75% of Austria’s GDP in 2006 and 3.5% of The Netherlands’ GDP in 2008), in Portugal, that value was 0.66%, 0.61% and between 0.4 and 0.8% in 2002, 2012, and 2018, respectively [13]. Nonetheless, in 2021, Portugal saw a 26.6% increase in volunteers registered in volunteering platforms [14].

There are two key stakeholders in the provision of voluntary support in mental health: the mental health professionals and the volunteers. The former identifies recovery needs, prescribes and recommends their patients to volunteering programmes, while the latter is the ‘fuel’ that is fundamental to the existence of those programmes [15].

The existing literature on volunteering focuses mainly on the motivations and characteristics of volunteers [16, 17] as well as its impacts and health benefits [18, 19], leaving a gap of knowledge of the views of mental health professionals and volunteers on volunteering in mental health.

This study aimed to fill that gap and explore and compare the views of these two key stakeholders in Portugal on volunteering in mental health care, focusing on the following research question: “What are the commonalities and differences of the views of mental health professionals and volunteers regarding volunteering in mental health care?”

## Methods

### Study design

This study was a qualitative secondary analysis of a subset of data taken from an international focus group study exploring two key stakeholders’ views (i.e., mental health professionals and volunteers) on volunteering in mental health [15]. The dataset from Portugal was the focus of this current investigation. Separate focus groups for each stakeholder group were conducted in Portugal between July 2016 and September 2017 in Porto. A full description of the methodological approach conducted in this study can be retrieved elsewhere [15].

### Participants

Mental health staff were recruited from the Hospital de Magalhães Lemos, a psychiatric hospital; volunteers were recruited from healthcare organisations, non-governmental organisations or volunteering and community associations in the region of Porto. Emails with information about the study were circulated to staff at Hospital de Magalhães Lemos and the volunteering organisations. Snowball sampling was used by inviting potential participants to share the invitation with their contacts. Information about the study was shared by the volunteering organisations’ websites, social media and e-mail. The focus groups with mental health professionals took place at the Hospital de Magalhães Lemos, while the ones with the volunteers were held at the University of Porto. Each lasted between 60 and 90 min and was conducted in Portuguese. A topic guide was used which focused on four overarching questions: the purpose of volunteers in the volunteering schemes, the character of the relationship between volunteers and people with mental disorders, the potential for the use of technology and the benefits and challenges of volunteering. Focus groups were audio recorded and transcribed verbatim. The study received ethical approval and written informed consent from all participants prior to the beginning of the group’s discussions [15] (see Ethics Statement).

### Data analysis

Materials obtained from the primary study included anonymised transcripts and topic guides, which were used to fully understand the context in which the data was collected and allow for immersion in the data. In this study a realist approach was adopted, whereby data collected was assumed to reflect something that is happening in the real world that exists independently of the researcher and participants, whilst acknowledging the role of social context [20]. Inductive reflexive thematic analysis was conducted following Braun and Clarke’s six-phase process [21]. All transcripts were uploaded to NVivo qualitative analysis software, V.12. First, initial codes—concise, explanatory or interpretive labels were created for information that may be relevant. Afterwards, the initial codes were reviewed in order to merge them according to shared meanings so they could form themes. This process was conducted by two researchers (JO and MPC), and themes were organised into main themes and subthemes. Subthemes representing commonalities were given the same name to denote similarity in the stakeholders’ views. For subthemes representing differences, it was ensured that each corresponded to a related fundamental point of contrast. In an iterative process, the names of the themes and sub-themes were refined to better respond to the research question of this analysis [21–23].

The research team for this qualitative secondary analysis comprised two researchers (JO, a male MSc student in psychology and MPC, a female clinical academic psychiatrist, who led the primary study).

Illustrative quotes were identified and reported next to each subtheme to support the analytical claims (Supplementary Table S1). This study reporting adhered to the Consolidated Criteria for Reporting Qualitative Research guidelines [24] (Supplementary Material 2).

## Findings

Four focus groups with mental health professionals were conducted with 16 participants. Most of them were women (n = 11, 68.8%) with an age range of 26–58 ($\overline{x} = 33.4$, Mdn = 28). Participants were mostly psychiatrists in training (n = 11, 68.8%) and the majority (n = 7, 70%) did not have experience in volunteering in mental health (Table 1). Two focus groups with volunteers were conducted with 12 participants. Most of them were women (n = 9, 75%) with an age range of 21–66 ($\overline{x} = 38.4$, Mdn = 38). Participants came from varying professional backgrounds and the majority did not have experience in volunteering in mental health (n = 10, 83.3%) (Table 2).

**Table 1:** Mental health professionals’ sociodemographics

Sociodemographics	Mental health professionals
Age	
Mean (SD)	33.4 (10.7)
Median (range)	28.0 (26–58)
Gender	
Female	11; 68.8%
Male	5; 31.3%
Professional background	
Psychiatrist	1; 6.3%
Psychiatrist in training	11; 68.8%
Psychologist	1; 6.3%
Nurse	1; 6.3%
Social worker	1; 6.3%
Occupational therapist	1; 6.3%
Experience in volunteering	
Yes	10; 62.5%
No	6; 37.5%
Experience in volunteering in mental health	
Yes	3; 30%
No	7; 70%

**Table 2:** Volunteers’ sociodemographics

Sociodemographics	Volunteers
Age	
Mean (SD)	38.4 (14.5)
Median (range)	38 (21–66)
Gender	
Female	9; 75%
Male	3; 25%
Professional background	
Dentist	3; 25%
Medical doctor	1; 8.3%
Nurse	1; 8.3%
Teacher	1; 8.3%
Special needs education teacher	1; 8.3%
Security	1; 8.3%
Receptionist	1; 8.3%
Street cleaner	1; 8.3%
Retired	2; 16.7%
Experience in volunteering in mental health	
Yes	2; 16.7%
No	10; 83.3%

Four overarching themes were identified (Table 3), from which, various subthemes emerged, which are presented below and summarised in each of the tables in Supplementary Table S1.

**Table 3:** Themes and subthemes

Themes	Mental health professionals	Volunteers
The nature of the volunteering relationship	The volunteer is not a health professional*	
	Defining boundaries for the volunteering relationship is important*	
	Both volunteer and patient should contribute financially to their joint activities*	
	Volunteering as a programmed relationship	Volunteering can be spontaneous
Volunteering has multiple aims	Volunteering to fight the stigma around mental health*	
	Volunteering to fight the social isolation of the patients*	
	Volunteering to empower the patient*	
	Volunteering to serve as a link to health professionals*	
	Volunteering to provide emotional support for the patients*	
	Volunteering to positively impact people with mental illness*	
	Volunteering also impacts the volunteers themselves*	
	Volunteering impacts every patient’s life	The impact on the patient’s life depends on their preferences
Technology has potential for volunteering	Technology complements communication between the patient and the volunteer*	
	Technology carries a risk of being misused by the patient*	
	Virtual communication is not preferable to face-to-face communication*	
	Technology to create new relationships	Technology serves not to create new relationships, but to maintain them
	Technology can be used to fight social isolation	Technology allows for anonymous conversations
Volunteering has its challenges	The stigma around mental health*	
	The training of the volunteers*	
	The emotional strength that the volunteer needs to have*	
	Ensuring that there is a structure of support*	
	The thoroughness of the selection process of the volunteers	Everyone can be a volunteer

*sub-themes for both mental health professionals and volunteers

### The nature of the volunteering relationship

One commonality between the two stakeholders with respect to the nature of the volunteering relationship was considering the volunteer not as a health professional, nor a professional of any kind, but as a human being that helps another human being with companionship and compassion.

“I think that there is a big separation here, that must be done, which is that the volunteer is not a health professional. The volunteer is doing (…) He is doing a voluntary work which, in itself does not have the objective of being profitable, it is almost of goodwill to do it.” (Mental Health Professionals Focus Group 04, Participant 03)

“While helping, we are attentive (…), sometimes one word is enough, one attitude is enough (…). We are more as human beings than as professionals of any sort” (Volunteers Focus Group 02, Participant 06)

Both stakeholders deemed it important to define boundaries for the regulation of the volunteering relationship, to serve as protection for both the volunteers and the patients, in order to prevent a relationship of dependence or abuse of personal boundaries.

“There has to be a limit. (…) We cannot forget about ourselves” (Volunteers Focus Group 01, Participant 02)

Mental health professionals and volunteers also agreed that with respect to financial contributions, both volunteers and patients should contribute equally and naturally to their joint activities (e.g., going to a coffee).

“I think that it largely depends on the moment, how the coffee was set, but I think that there is no payment obligation on one part or the other, or to be divided, people at the end, both parts figure it out naturally and I think nobody will take offense to whatever decision is made.” (Volunteers Focus Group 01, Participant 04)

“I think that the patient should be involved in that payment, and the activities should be chosen according to the socioeconomic status of the patient, it is not necessary to do expensive activities.” (Mental Health Professionals Focus Group 01, Participant 03)

However, mental health professionals and volunteers differed in their perspective of the definition of the volunteering relationship. For the former, volunteering can be a programmed relationship, in that it is structured and professional. For the latter, volunteering can be a spontaneous relationship, in that it can be informal and even exist daily.

“It is an organized thing, isn’t it? It’s not like (…) a friendship in the normal way. It is something that is “programmed”. So, it must also have some rules. What is important is that people get to know both parties and get to know them early on. The patient needs to know what he is going to count on, and what kind of relationship it is, and the volunteer needs to know what his role is. To know their roles, both.” (Mental Health Professionals Focus Group 04, Participant 04)

“I think that it is important to understand the concept of volunteering, which has already been discussed here, and I think that we all need to understand that volunteering is not official. I think that we should also look at what we do in our day-to-day and even at what other people do in their day-to-day that most of the time is a lot. Other people I believe they are giving a lot of themselves to help people even if not in an official volunteering context, and that should be taken into account as volunteering that helps, and I think that helps society a lot and helps everyone else.” (Volunteers Focus Group 01, Participant 04)

### Volunteering has multiple aims

Participants from both stakeholders’ groups generally described various aims of volunteering programmes, such as fighting the stigma around mental health, fighting isolation, empowering the patient, serving as a link to health professionals, providing emotional support for the patients and positively impacting both people with mental illness and the volunteers themselves.

“Because people with mental illness are seen as if they were almost as vermin, animals, it is nothing like that, they are people like us, so, I think that [the goal] is a little to break (…) that myth.” (Volunteers Focus Group 01, Participant 02)

“It would be a lot to fight isolation too, right? We know that some of these people end up being very isolated.” (Mental Health Professionals Focus Group 02, Participant 01)

“And I think that volunteering even enriches us a lot inside. Volunteering enriches us a lot.” (Volunteers Focus Group 01, Participant 03)

“And I find n people who would benefit, people with psychopathologies who would benefit from being volunteers, I think these [volunteering] centres should exist, even if we weren’t sure that they would benefit the patients, but we would know that it would benefit the volunteers.” (Mental Health Professionals Group 01, Participant 02)

The perspectives of mental health professionals and volunteers differed on the impact that volunteering has on the patient’s life, as the former felt that it has an impact even if an indirect impact, and the latter believed that the impact depends on patients’ preferences.

“Imagine, [the patient] is with a volunteer. Maybe therapy adherence is going to improve, hence, the heterologous activity will also be lower, we hope, right? That is, it would be an indirect measure of the impact of the volunteer in the life of that patient.” (Mental Health Professionals Group 03, Participant 01)

“The impact? It also depends, I think it depends on the people who receive it, there are some people who like it, people who don’t. People who like the company, people who don’t like to share things, people who don’t like it.” (Volunteers Focus Group 01, Participant 04)

### Technology has potential for volunteering

Mental health professionals and volunteers mentioned that although technology has the potential to complement communication between the patient and the volunteer, it carries a risk of being misused. For some participants in both stakeholder groups, virtual communication is not preferable over face-to-face communication.

“To make a phone call, or for example via Skype, let’s imagine that… I am a volunteer and for some time I am not here, one week or two, whatever it is, [to] keep in touch with the person, to be able to talk, to keep up to date with new situations, I think it is important.” (Volunteers Focus Group 01, Participant 02)

“And who guarantees that it is being used correctly?” (Mental Health Professionals Group 02, Participant 02)

“After all, if we see it as it is, these are people with difficulties in verbal communication, that is, face-to-face communication can be much better for them, and we are not there sending stimuli that they may even misinterpret (…)” (Mental Health Professionals Focus Group 01, Participant 01)

Mental health professionals and volunteers differed in that the former believes that technology for volunteering programmes could help to create new relationships, while the latter believes that it serves to maintain relationships, not to create them. Mental health professionals mentioned that technology could be used to fight isolation, while volunteers mentioned that one of the best uses for technology is that it would allow for anonymous conversations, maintaining the privacy and improving the patient’s trust and freedom to express themselves.

“Imagine, for example, a platform, an app that all volunteers and all patients have access to and can communicate through a chat, for example. Or a forum, something like that. Everyone can exchange ideas and from there, perhaps, create new relationships.” (Mental Health Professionals Focus Group 04, Participant 01)

“Forming new relationships is possible through technology, but I think it is essentially important to maintain a relationship. I think that, for me, I don’t usually create relationships over Skype. I think it is a bit, I don’t know, it’s weird for me.” (Volunteers Focus Group 01, Participant 04)

“Sometimes a person can talk more freely and vent a lot more and listen a lot more if they don’t know who it is [through technology] (…)” (Volunteers Focus Group 01, Participant 01)

“Facebook is trending, maybe used well it could be a method of (…) company, sharing, talking with someone, not feeling so lonely, I highlight once again, used well.” (Mental Health Professionals Group 02, Participant 03)

### Volunteering has its challenges

Although every focus group acknowledged the potential and benefits of volunteering programmes, one of the main points of discussion were the barriers and hurdles that volunteering still needs to address. Both stakeholders’ groups identified the presence of stigma around mental health as one of the main challenges of volunteering, describing mental illness as still marginalised in the Portuguese society.

“(…) the obstacles have to do with the lack of understanding of mental illness.” (Mental Health Professionals Focus Group 02, Participant 03)

“My biggest challenge is, above all, to reach the stakeholders, to make them understand that mental health and homeless people are human beings who deserve all the dignity (…)” (Volunteers Focus Group 02, Participant 01)

The need to train volunteers as well as the existence of a structure of support to guarantee that training, was also an important challenge mentioned by both mental health professionals and volunteers. This was suggested as a way for volunteers to maintain a helping relationship with people with mental illness, for which communication and critical thinking skills were perceived as essential.

“And there must really exist a structure behind to really do this training, this selection, (…) to really work on these questions and these social skills, the isolation (…)” (Mental health Professionals Group 02, Participant 01)

“And training, yes, I think that is important because, there it is, I have no idea how to deal with these people.” (Volunteers Focus Group 01, Participant 04)

Both stakeholders mentioned the emotional strength that the volunteer requires to deal with people with mental illness. Whilst the mental health professionals felt that the selection process must have rigorous criteria, volunteers felt that everyone can be a volunteer.

“Because I think that to be a volunteer you need to have a heart of the size of the world.” (Volunteers Focus Group 01, Participant 01)

“And the selection itself. I believe that (…) there must be a really careful and strict selection process [as possible] to (…) be useful, so that there is a beneficial and not a prejudicial interaction.” (Mental health Professionals Group 02, Participant 01)

“(…) I think that the opportunity should be extended (…) to every person that has the availability to do it (…)” (Volunteers Focus Group 01, Participant 04)

## Discussion

Among the four main themes, there was consistency in the number of commonalities and differences identified. In only one theme “Technology has potential for volunteering” there were more than one point of difference between stakeholders. The poor tradition of volunteering in Portugal may have contributed to the lack of diverse and disparate perspectives on this topic due to a lack of conversation and discussion regarding volunteering, which may be undermining the quality of public opinion.

Across all themes, there was a notorious difference between mental health professionals and volunteers in Portugal, as the former leaned onto a rigorous and structured view of volunteering and the latter took a more flexible, phenomenological and humanitarian stance. This distinct pattern may be a manifestation of the impact of the experience of volunteering on the stakeholders’ perspectives. In our data, mental health professionals took a more goal-oriented and mental health rehabilitation approach with patients, and volunteers reflected on their own personal and direct experiences with volunteering, which represented the will to connect with the patient and to feel personally fulfilled.

Both stakeholders agreed on the complementary nature of volunteering, as it was viewed as a relationship that had the potential to address the need for social inclusion as well as for empowerment and emotional support for the patient. Additionally, the fight against the stigma around mental health was a trans-thematic concern for mental health professionals and volunteers, as they felt that volunteering could help in that fight.

Concerning technology, although it was seen with great potential, risks were anticipated by both stakeholders. The perspectives on virtual communication were a mix of understanding its ability to diminish isolation, complement communication, and the risk of being misused or a reluctance in using virtual over face-to-face communication.

### Strengths and limitations

This is the first study to compare the views of mental health professionals and volunteers in Portugal. Another strength is that the research objectives of this secondary qualitative study are closely aligned with the original research, supporting the relevance of the data for this secondary analysis [25]. Secondary qualitative research that is conducted without direct involvement in the primary study can lead to a poor understanding of perspectives in their context. This was not the case in this study, since the lead researcher of the primary study, has been one of the researchers involved in this qualitative secondary analysis. In addition, the other researcher has, as recommended [26], familiarised and immersed themselves with the original data as well as with additional materials such as coding information and relevant literature produced by the primary researcher. Moreover, the primary study’s lead researcher provided direct supervision to the second researcher, which allowed for a full understanding of the data.

Secondly, the focus groups were divided by stakeholders to mitigate the potential risk of having uneven group interactions between mental health professionals and volunteers. However, variations within groups must be acknowledged. In mental health professionals’ groups, there was a 3:7 ratio of experience and no experience in volunteering in mental health, respectively; whilst in the groups of volunteers, participants had different job roles (with some being health professionals) and there was a 2:10 ratio of experience and no experience in volunteering in mental health. This may translate into some variations in their characteristics and experiences, which may impact their perspectives on the discussed topic.

Finally, since this study was conducted with mental health professionals working in inpatient and outpatient services from one of the largest mental health hospitals in Portugal, the findings may not be directly applicable to other contexts.

### Comparison with the literature

The first theme highlights the paradoxical nature of the volunteering relationship, as it can be seen as spontaneous or programmed and that the volunteer is not a health professional, but the relationship still needs boundaries. This spectrum of the volunteering relationship has already been described [10] as two ends of the spectrum: at the one end is a professional relationship and at the other, a relationship closer to a friendship.

The second theme highlights a wide range of aims associated with volunteering, ranging from the impact that volunteering can have on both patients and volunteers, to empowering the patient and fighting the stigma. Cassidy and colleagues [27] have found similar results in a thematic analysis of motivations and experiences of volunteers, in that personal growth and altruism were found to be motivators for volunteering (e.g., having new experiences and contributing to society) as well as the impact of “doing things”, which focused on giving advice and getting out, which may infer onto the empowerment of the patient. Furthermore, findings from a study on volunteering and its impact on hospital performance suggested that volunteers offer significant cost savings to hospitals and enhance patient satisfaction scores [28].

The third theme highlights the potential of technology, and it comprises its perceived advantages and risks. It focused on the debate between virtual and face-to-face communication, with opposing views coming from both groups. However, stakeholders agree that technology should be complementary and that it carries a risk of being misused. This duality goes in line with results from a survey of the preferences of patients with severe mental illness in contacting with a volunteer, as results reported that 57.6% and 37.1% of patients were interested in getting face-to-face and digital volunteering support, respectively [29]. The use of technology in the healthcare settings is still the subject of an ongoing discussion, with findings from a systematic review suggesting that health workers have a mixed, complex and nuanced view of its use [30]. Furthermore, this Portuguese avoidant and fearful perspective on technology have already been demonstrated with data from the Eurostat that put Portugal below the EU average on technological implementation in businesses after COVID-19 [31].

The last theme highlights that there are challenges in volunteering in mental health care that need to be overcome. It focuses on several matters that the volunteering organization, the patient and society itself need to address, such as the stigma around mental illness or ensuring that there is a structure that can serve as an emergency net for the volunteer. This focus on the inclusion and stigma around mental health may reflect the mental health paradigm in Portuguese society, which is characterized by, besides other things, the presence of stigma [32]. Similarly, a rapid review by Southby et al. [33] reported that volunteers have a lack of appropriate support, volunteer management, time, and financial support and feel that there is a poor perception of volunteering as well as that it is a stigmatising/exclusionary context.

### Implications of the findings for future practice and research

These findings illustrate some Portuguese perceptions of volunteering in mental health by two key stakeholder groups from the north part of Portugal, the mental health professionals and the volunteers themselves, and may be used to inform the structuring of current and new volunteering programmes. Future research could collect the stakeholders’ views based on other regions in Portugal, including both urban and rural areas, and gather as well the perspectives of patients in mental health services.

Since Portugal has a poor volunteering tradition, these results may aid in the planning and implementation of new volunteering initiatives, and advice on health policies. Future studies should dwell on the Portuguese society’s views on volunteering, to mitigate some of the challenges identified, of which addressing stigma is emphasised.

Furthermore, since there was a lack of direct experience in volunteering in mental health within the volunteer group, it becomes unclear what understanding volunteers had of mental illness and mental health care, which could influence their perspectives. Future research should consider sampling volunteers with direct experience in this field.

## Conclusions

In Portugal, mental health professionals and volunteers regard programmes of volunteering in mental health care as a significant opportunity to positively impact both patients and volunteers. Overall, stakeholders’ perspectives suggested a need for structured recruitment and support, training, defining boundaries and fighting the stigma of mental illness. However, promoting volunteering opportunities in mental health in Portuguese society may be the primary goal, especially in light of the current paucity of such initiatives.

## Supplementary Information

Below is the link to the electronic supplementary material.

Supplementary file 1 (DOCX 32 KB)

Supplementary file 2 (DOCX 58 KB)

## Data Availability

All data analysed during the current study are available from the corresponding author on reasonable request.
