# Supplementary material for: Views on volunteering in mental health: a focus group study with mental health professionals and volunteers in Portugal
Source: Discov Ment Health. 2023 Jun 22;3(1):12. doi: 10.1007/s44192-023-00038-1 (PMC10501020; doi:10.1007/s44192-023-00038-1)
Supplement: Supplementary file 1 — (DOCX 32 KB) [file 44192_2023_38_MOESM1_ESM.docx]

| Supplementary table 1 - Themes, subthemes and illustrative quotes | | | |
| --- | --- | --- | --- |
| The nature of the volunteering relationship | | | |
|  | **Mental Health Professionals** | | **Volunteers** |
| Commonalities | **The volunteer is not a health professional** | | |
|  | “I think that there is a big separation here, that must be done, which is that the volunteer is not a health professional. The volunteer is doing… He is doing voluntary work which, in itself does not have the objective of being profitable, it is almost of goodwill to do it.” *(Mental Health Professionals Focus Group 04, Participant 03)* | | “While helping, we are attentive… sometimes one word is enough, one attitude is enough (…). We are more as human beings than as professionals of any sort.” *(Volunteers Focus Group 02, Participant 06)* |
|  | **Defining boundaries for the volunteering relationship is important** | | |
|  | “More than the training, more than whether the person is genuinely good at volunteering or not, I think that the most relevant and difficult thing is generally to set boundaries. A time limit, boundaries of confidentiality, limits in exchanging contacts, these are generally the limits for me, it is the most important thing to define.” *(Mental Health Professionals Group 02, Participant 04)* | | “There has to be a limit. (…) We cannot forget about ourselves.” *(Volunteers Focus Group 01, Participant 02)* |
|  | **Both volunteer and patient should contribute financially to their joint activities** | | |
|  | “I think that the patient should be involved in that payment, and the activities should be chosen according to the socioeconomic status of the patient, it is not necessary to do expensive activities.” *(Mental Health Professionals Focus Group 01, Participant 03)* | | “I think that it largely depends on the moment, how the coffee was set, but I think that there is no payment obligation on one part or the other, or to be divided, people at the end, both parts figure it out naturally and I think nobody will take offense to whatever decision is made.” *(Volunteers Focus Group 01, Participant 04)* |
| Differences | **Volunteering as a programmed relationship** | | **Volunteering can be spontaneous** |
|  | “It is an organized thing, isn’t it? It’s not like (…) a friendship in the normal way. It is something that is “programmed”. So, it must also have some rules. What is important is that people get to know both parties and get to know them early on. The patient needs to know what he is going to count on, and what kind of relationship it is, and the volunteer needs to know what his role is. To know their roles, both.” *(Mental Health Professionals Focus Group 04, Participant 04)* | | “I think that it is important to understand the concept of volunteering, which has already been discussed here, and I think that we all need to understand that volunteering is not official. I think that we should also look at what we do in our day-to-day and even at what other people do in their day-to-day, that most of the time is a lot. Other people I believe they are giving a lot of themselves to help people even if not in an official volunteering context, and that should be taken into account as volunteering that helps, and I think that helps society a lot and helps everyone else.” *(Volunteers Focus Group 01, Participant 04)* |
| Volunteering has multiple aims | | | |
|  | **Mental Health Professionals** | | **Volunteers** |
| Commonalities | **Volunteering to fight the stigma of mental health** | | |
|  | “(…) In fact, I think that [volunteering] can play an important role in fighting the stigma, which is still something that worries us a lot and I think that even the volunteers themselves, then in their community and telling what happens in these relations can also promote the fighting of the stigma. I think this could be important.” *(Mental Health Professionals Group 04, Participant 02)* | “Because people with mental illness are seen as if they were almost as vermin, animals, it is nothing like that, they are people like us, so, I think that [the goal] is a little to break (…) that myth.” *(Volunteers Focus Group 01, Participant 02)* | |
|  | **Volunteering to fight the social isolation of the patients** | | |
|  | “It would be a lot to fight isolation too, right? We know that some of these people end up being very isolated.” *(Mental Health Professionals Focus Group 02, Participant 01)* | “(…) but I think it is more about the company and often these people do not realize of the situations they are in, or if they realize, they can feel lonely and different from others, and I think keeping these people company also helps them to feel better and I think that is essentially what it is, it is helping people feel better about themselves.” *(Volunteers Focus Group 01, Participant 04)* | |
|  | **Volunteering to empower the patient** | | |
|  | “I think to give the patient a standard of mental health. And in principle if that person, the volunteer does not have psychopathology, it will be an example of a healthy conversation, isn’t it? A person that can [give], some empowerment to the patient, isn’t it? Not always talking about those negative things, that a patient with mental illness perhaps, a lot of time talks about, a dark vision of the world for example. I think that is it, to give a healthier perspective of the world.” *(Mental Health Professionals Focus Group 01, Participant 04)* | “Things that, in addition to the social component which in this case is also more prevalent on the side of the health professionals, our main role ends up being a little educational, trying to teach, and perhaps more than once, because sometimes we come across with people that do not understand it on the first, second or third, and we need to have the capacity to turn the situation around, to understand the person’s limitations, to help her to have some extra care for herself.” *(Volunteers Focus Group 02, Participant 04)* | |
|  | **Volunteering to serve as a link to health professionals** | | |
|  | “We can also have a person that can report, for example, when there is any problem. Even reporting to the professionals if there is any sign of decompensation. To exist a link, also, that can promote that health services always know whenever something happens to the patient.” *(Mental Health Professionals Focus Group 04, Participant 02)* | “Even for most times, as it happens in our case, reporting cases that people are in the streets, with mental health issues and that don’t know to whom they can call because they are lost, they don’t even have any discerning capacities, to anything.” *(Volunteers Focus Group 02, Participant 5)* | |
|  | **Volunteering to provide emotional support for the patients** | | |
|  | “I think that besides all that, all that has been said, it turns out to be important too, as a safe haven in crisis situations. Not only for the volunteer that goes to be with the patient and that helps him to manage the illness and medication, but also in the opposite direction, the patient has a person to look for, which ends up replacing, many times, the access to health care, because the patient often goes to the emergency services with topics and issues that are not directly related to the illness.” *(Mental Health Professionals Focus Group 04, Participant 04)* | “I think the main role is to… be as if it were a help in terms of, as if it were a caregiver for that person, it may not be, for example, in the principle of dealing with a person, but it is like an emotional and physical support, often for the people, it is not to be, it is not being there for everything that is necessary, but on the things that the being able to help, make that difference.” *(Volunteers Focus Group 01, Participant 01)* | |
|  | **Volunteering to positively impact people with mental illness** | | |
|  | “And I find *n* people who would benefit, people with psychopathologies who would benefit from being volunteers, I think these [volunteering] centres should exist, even if we weren’t sure that they would benefit the patients, but we knew that they would benefit the volunteers.” *(Mental Health Professionals Group 01, Participant 02)* | | “And the person who is being helped, if we are doing it, if we are helping they will, they may feel it or they may not feel it, but they will end up being helped, I think that regardless of whether they are conscious of that or not, I think they have always benefits. I think that it is important.” *(Volunteers Focus Group 01, Participant 02)* |
|  | **Volunteering also impacts the volunteers** | | |
|  | “And they [people with mental health problems] are a population which you cannot properly reach, and we come into contact with a very different reality, that is, for the volunteers, they are contacting with a reality that they do not know, these are new experiences for the patients, but experiences for the volunteers.” *(Mental Health Professionals Focus Group 01, Participant 01)* | | “And I think that volunteering even enriches us a lot inside. Volunteering enriches us a lot.” *(Volunteers Focus Group 01, Participant 03)* |
| Differences | **Volunteering impacts every patient’s life** | | **The impact on the patient’s life depends on their preferences** |
|  | “Imagine, [the patient] is with a volunteer. Maybe therapy adherence is going to improve, hence, the heterologous activity will also be lower, we hope, right? That is, it would be an indirect measure of the impact of the volunteer in the life of that patient.” *(Mental Health Professionals Group 03, Participant 01)* | | “The impact? It also depends, I think it depends on the people who receive it, there are people who like it, people who don’t. People who like company, people who don’t like to share things, there are people who don’t like.” *(Volunteers Focus Group 01, Participant 04)* |
| Technology has potential for volunteering | | | |
|  | **Mental Health Professionals** | | **Volunteers** |
| Commonalities | **Technology complements communication between the patient and the volunteer** | | |
|  | “Complementing face-to-face [communication]. It is not like it happens nowadays, which is almost replacement.” *(Mental Health Professionals Group 02, Participant 01)* | | “To make a phone call, or for example via Skype, let’s imagine that… I am a volunteer and for some time I am not here, one week or two, whatever it is, [to] keep in touch with the person, to be able to talk, to keep up to date with new situations, I think it is important.” *(Volunteers Focus Group 01, Participant 02)* |
|  | **Technology carries a risk of being misused by the patient** | | |
|  | “And who guarantees that it is being used correctly?” *(Mental Health Professionals Group 02, Participant 02)* | | “Like everything in life, with moderation, right? (…) If there is no control, there is no moderation.” *(Volunteers Focus Group 02, Participant 01)* |
|  | **Virtual communication is not preferable over face-to-face communication** | | |
|  | “After all, if we see it as it is, these are people with difficulties in verbal communication, that is, face-to-face communication can be much better for them, and we are not there sending stimuli that they may even misinterpret (…)” *(Mental Health Professionals Focus Group 01, Participant 01)* | | “I think that face-to-face [communication] helps more, but over the phone, it also helps.” *(Volunteers Focus Group 01, Participant 03)* |
| Differences | **Technology to create new relationships** | | **Technology serves not to create new relationships, but to maintain them** |
|  | “Imagine, for example, a platform, an app that all volunteers and all patients have access to and can communicate through a chat, for example. Or a forum, something like that. Everyone can exchange ideas and from there, perhaps, create new relationships.” *(Mental Health Professionals Focus Group 04, Participant 01)* | | “Forming new relationships is possible through technology, but I think it is essentially important to maintain a relationship. I think that, for me, I don’t usually create relationships over Skype. I think it is a bit, I don’t know, it’s weird for me.” *(Volunteers Focus Group 01, Participant 04)* |
|  | **Technology can be used to fight social isolation** | | **Technology allows for anonymous conversations** |
|  | “Facebook is trending, maybe used well it could be a method of (…) company, sharing, talking with someone, not feeling so lonely, I highlight once again, used well.” *(Mental Health Professionals Group 02, Participant 03)* | | “Sometimes a person can talk more freely and vent a lot more and listen a lot more if they don’t know who it is [through technology] (…)” *(Volunteers Focus Group 01, Participant 01)* |
| Volunteering has its challenges | | | |
|  | **Mental Health Professionals** | | **Volunteers** |
| Commonalities | **The stigma around mental health** | | |
|  | “(…) the obstacles have to do with the lack of understanding of mental illness.” (Mental Health Professionals Focus Group 02, Participant 03) | | “My biggest challenge is, above all, to reach the stakeholders, to make them understand that mental health and homeless people are human beings who deserve all the dignity (…)” *(Volunteers Focus Group 02, Participant 01)* |
|  | **The training of the volunteers** | | |
|  | “It is essential … I think that to match people with the mentally ill without explaining the basic what a mental illness is would not work.” *(Mental Health Professionals Focus Group 02, Participant 03)* | | “And training, yes, I think that is important because, there it is, I have no idea how to deal with these people.” *(Volunteers Focus Group 01, Participant 04)* |
|  | **The emotional strength that the volunteer needs to have** | | |
|  | “It is not so much the person’s stigma, but it is the person’s capacity or not to handle one of those situations.” *(Mental Health Professionals Group 02, Participant 03)* | | “Because I think that to be a volunteer you need to have a heart of the size of the world.” *(Volunteers Focus Group 01, Participant 01)* |
|  | **Ensuring that there is a structure of support** | | |
|  | “And there must really exist a structure behind to really do this training, this selection, (…) to really work on these questions and these social skills, the isolation (…)” *(Mental health Professionals Group 02, Participant 01)* | | “(…) There always has to be an image of support, someone central, there has to be a bond that shows that that person may not be there on that day, but there is another support, there is a backup, and there is something central here that organizes everything and guarantees that there is a connection.” *(Volunteers Focus Group 02, Participant 04)* |
| Differences | **The thoroughness of the selection process of the volunteers** | | **Everyone can be a volunteer** |
|  | “And the selection itself. I believe that (…) there must be a really careful and strict selection process [as possible] to (…) be useful, so that there is a beneficial and not a prejudicial interaction.” *(Mental health Professionals Group 02, Participant 01)* | | “(…) I think that the opportunity should be extended (…) to every person that has the availability to do it (…)”  *(Volunteers Focus Group 01, Participant 04)* |
